# Ultrasound Guidance for Renal Tract Access and Dilation Reduces Radiation Exposure during Percutaneous Nephrolithotomy

**DOI:** 10.1155/2016/3840697

**Published:** 2016-03-02

**Authors:** Thomas Chi, Selma Masic, Jianxing Li, Manint Usawachintachit

**Affiliations:** ^1^Department of Urology, University of California, San Francisco, 400 Parnassus Avenue, Suite A610, P.O. Box 0330, San Francisco, CA 94143, USA; ^2^Department of Urology, Tsinghua Changgung Hospital, Beijing 100034, China; ^3^Division of Urology, Department of Surgery, Faculty of Medicine, Chulalongkorn University, King Chulalongkorn Memorial Hospital, Rama IV Road, Patumwan, Bangkok 10330, Thailand

## Abstract

*Purposes*. To present our series of 38 prone percutaneous nephrolithotomy procedures performed with renal access and tract dilation purely under ultrasound guidance and describe the benefits and challenges accompanying this approach.* Methods*. Thirty-eight consecutive patients presenting for percutaneous nephrolithotomy for renal stone removal were included in this prospective cohort study. Ultrasonographic imaging in the prone position was used to obtain percutaneous renal access and guide tract dilation. Fluoroscopic screening was used only for nephrostomy tube placement. Preoperative, intraoperative, and postoperative procedural and patient data were collected for analysis.* Results*. Mean age of patients was 52.7 ± 17.2 years. Forty-five percent of patients were male with mean BMI of 26.1 ± 7.3 and mean stone size of 27.2 ± 17.6 millimeters. Renal puncture was performed successfully with ultrasonographic guidance in all cases with mean puncture time of 135.4 ± 132.5 seconds. Mean dilation time was 11.5 ± 3.8 min and mean stone fragmentation time was 37.5 ± 29.0 min. Mean total operative time was 129.3 ± 41.1. No patients experienced any significant immediate postoperative complication. All patients were rendered stone-free and no additional secondary procedures were required.* Conclusions*. Ultrasound guidance for renal access and tract dilation in prone percutaneous nephrolithotomy is a feasible and effective technique. It can be performed safely with significantly reduced fluoroscopic radiation exposure to the patient, surgeon, and intraoperative personnel.

## 1. Introduction

Percutaneous nephrolithotomy (PCNL) is a procedure commonly performed by urologists worldwide. Since its first introduction by Fernström and Johansson in 1976, it has become the mainstay surgical treatment for renal stones larger than 2 centimeters and those refractory to shockwave therapy [1]. Nevertheless, one concern related to this procedure is the possible long-term effects of ionizing radiation exposure sustained by the surgeon, medical personnel, and patient during the operation which is usually guided by fluoroscopic imaging [2, 3]. Numerous studies have shown that occupational exposure dose during fluoroscopic procedures can be kept within safe limits with routine use of protective aprons and thyroid shields, but some degree of radiation exposure to intraoperative personnel can still be detected [4–6]. One main factor influencing exposure is fluoroscopic screening time, which is significantly affected by total stone burden and the need for multiple accesses [7].

The utilization of ultrasound (US) can obviate the need for ionizing radiation exposure intraoperatively and provide a reliable method for the localization of renal stones. It can help surgeons create an appropriate access into the collecting system via a posterior calyx, guide tract dilation, and even confirm stone clearance after the procedure is completed. In addition, it can be an ideal imaging modality for special patient populations, including pregnant [8] and pediatric patients [9]. Others have demonstrated the feasibility and usefulness of complete ultrasound guidance in PCNL using a supine position [10–12].

In this study, we report our experience with ultrasound guidance for renal access and tract dilation in prone PCNL. We also evaluate the feasibility of this technique in the minimally dilated collecting system.

## 2. Patients and Methods

This was a prospective observational cohort study completed at two academic medical centers, University of California, San Francisco (UCSF), and San Francisco General Hospital (SFGH). In May of 2014, the operative surgeon for this study (TC) changed his approach for renal puncture from fluoroscopy guided access to ultrasound-guided access. Prior to that, PCNL had been performed under fluoroscopy guidance alone. After institutional IRB was obtained, clinical data for all patients presenting between March and August 2015 requiring treatment with PCNL was collected. Inclusion criteria for this study were (1) patients with renal or proximal ureteral stone, (2) age greater than 18 years. No patients were excluded from this analysis during the study time frame. Procedures performed during this time period utilized ultrasound guidance for all steps of the renal access and dilation. All procedures were performed by a single surgeon (TC) and perioperative data was collected prospectively for these analyses.

Preoperatively, complete blood count, serum creatinine, and demographic data were obtained from all patients. Non-contrast CT was used to determine stone characteristics. Ultrasound-guided cases were compared to a control cohort of cases done with fluoroscopic guidance by the same surgeon prior to the adoption of ultrasound guidance and matched for stone size to the ultrasound-guided group. Student's *t*-test and Chi-square test were used to compare differences between the two groups and statistical analyses were performed using Stata/IC version 13.1 (StataCorp, Texas, USA). Data are expressed as mean ± standard deviation or percentage with a significance level of *p* < 0.05.

**Figure 1:**
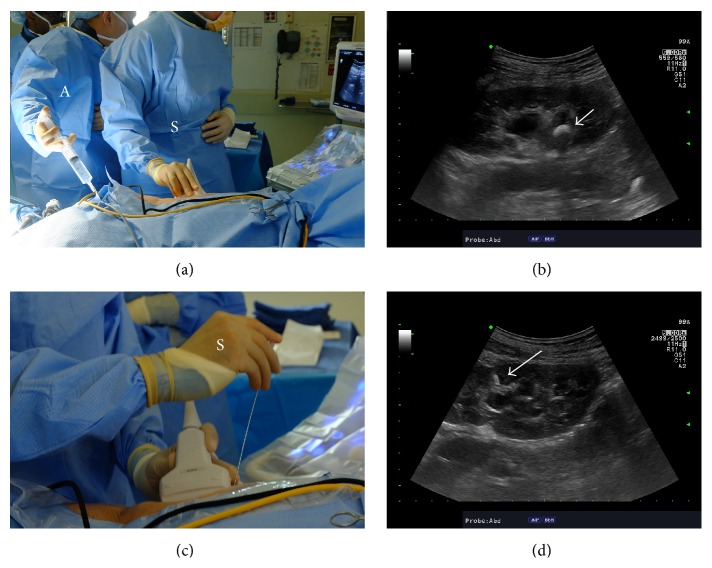
Establishing renal access using ultrasound guidance. (a) The operative surgeon (S) holds the ultrasound probe and the assistant (A) holds a syringe attached to ureteral catheter for normal saline infusion if needed. (b) Ultrasonographic image of the kidney along its longitudinal axis demonstrating the stone in the renal pelvis (white arrow) within a mildly hydronephrotic collecting system. (c) During the needle insertion, the operative surgeon (S) holds both the ultrasound probe and the needle to perform the puncture. For hand positioning, the nondominant hand holds the ultrasound probe while the dominant hand holds the needle. (d) The needle can be visualized (white arrow) entering the collecting system through upper pole calyx in this case.

**Figure 2:**
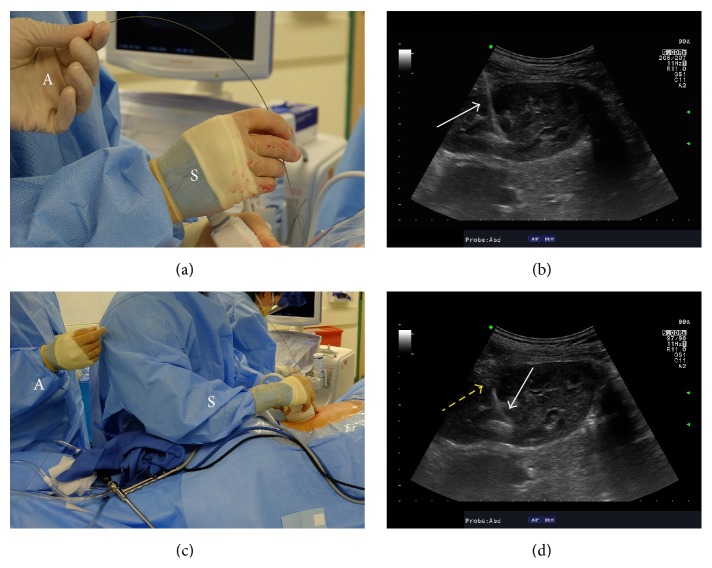
Introduction of working wire and dilators using ultrasound guidance. (a) A J-tip coaxial wire is inserted through the needle after its tip is confirmed in the collecting system. The operative surgeon (S) controls the needle with dominant hand and holds the ultrasound probe with the nondominant hand while the assistant (A) controls the wire. (b) Ultrasonographic image of the kidney demonstrating the wire (white arrow) in the collecting system after needle withdrawal. Its appearance is highly echogenic and prominent. (c) For fascial dilation, a 10-French fascial dilator is passed over the wire. Again, the operative surgeon (S) holds the ultrasound probe and controls the dilator while the assistant (A) controls the wire. (d) As the fascial dilator passes over the working wire, the wire is seen very clearly as an echogenic line (white arrow). The dilator obscures the view of the wire and this interface of the echogenic wire and the area where the wire disappears from view is the tip of the fascial dilator (yellow arrow).

**Figure 3:**
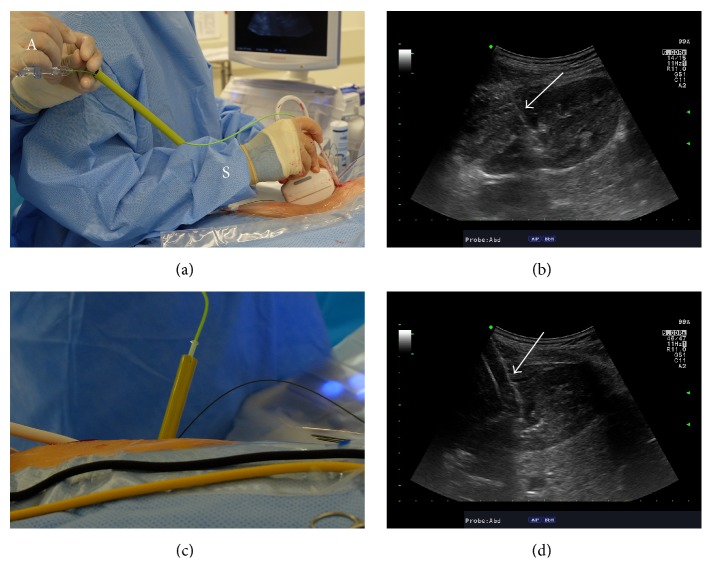
Tract dilation with high-pressure balloon under ultrasound guidance. (a) The deflated balloon dilator is inserted over a working wire and for this step, the operative surgeon (S) controls the ultrasound probe and distal end of the balloon while the assistant (A) controls the wire on the proximal end of the balloon dilator. (b) Ultrasonographic image of the kidney along its longitudinal axis demonstrates that the tip of the deflated balloon dilator (white arrow) is difficult to visualize and differentiate from the wire. (c) The sheath has been inserted over the inflated balloon dilator that is subsequently withdrawn. (d) The inflated balloon (white arrow) can be readily seen with ultrasound imaging.

After induction of general anesthesia, an open-ended 5-French ureteral catheter was inserted into the ipsilateral ureter for approximately 20 centimeters under cystoscopic guidance from a supine position with the patient on the transport gurney. Patients were then moved to a prone position and safely secured to the operative table. Percutaneous renal access was obtained by the operative surgeon using ultrasound guidance without a needle guide. We used a 3.5-MHz convex abdominal transducer (Hitachi Aloka Medical) to localize the stone position as well as an ideally suited posterior calyx for puncture (Figures 1(a) and 1(b)). An 18-gauge Echotip needle (Cook Medical) was advanced under real-time ultrasound monitoring (Figures 1(c) and 1(d)). In the absence of hydronephrosis, saline was injected retrograde through the ureteral catheter to dilate the collecting system for easier imaging though this was not routinely done for every patient. Entry into the collecting system was confirmed with either aspiration or efflux of urine through the puncture needle or clear visualization of the needle tip within the urinary space or touching the renal stone (Figure 1(d)). After entry into the collecting system was confirmed, a J-tip coaxial guide wire (Bard Medical) was inserted into the renal pelvis or down the proximal ureter, using ultrasound imaging to visualize the wire passing into the collecting system (Figures 2(a) and 2(b)). We then sequentially passed a 10-French fascial dilator and a safety wire introducer over the wire. The tip of each instrument was visualized with ultrasound imaging entering the collecting system to prevent collecting system perforation. The wire appeared with a very bright echogenic signal ultrasonographically, while the dilator and safety wire introducer were not echogenic. Their advancement over the wire could be visualized as they obscured the echogenic appearance of the wire. By watching for disappearance of the wire, the exact position of the tip of each dilator was visualized (Figures 2(c) and 2(d)). Using the safety wire introducer, a second safety wire was advanced into the collecting system and imaged as it passed into the collecting system. The balloon was then imaged passing into the collecting system (Figures 3(a) and 3(b)) over one of the wires and a working tract was dilated with a high-pressure balloon dilator (BARD X-Force, Bard Medical) using ultrasonography. Then, either a 24- or 30-French sheath was advanced over the inflated balloon (Figures 3(c) and 3(d)). After removal of the balloon, nephroscopy was performed with a 20.8- or 27-French rigid offset nephroscope (Richard Wolf Medical). Stone fragmentation was accomplished using an ultrasonic CyberWand lithotripter (Olympus America). Flexible nephroscopy was performed to look for residual fragments and holmium laser lithotripsy via a flexible cystoscope was used if needed for their removal. Stone clearance was confirmed intraoperatively with ultrasound imaging and nephroscopy. At the end of the procedure, a nephrostomy tube was placed in all patients under limited fluoroscopic screening. We routinely used a 10-French Cope loop catheter (Cook Medical) for this purpose.

Intraoperatively, fluoroscopic screening was used only for nephrostomy tube placement, positional confirmation, and readjustment using a mobile multidirectional C-arm fluoroscopy unit with an under table X-ray. Renal access puncture time (defined as the time elapsed from initial renal ultrasonographic imaging to successful placement of the needle into the collecting system), tract dilation time with US guidance (defined as the time elapsed from insertion of the wire into the collecting system to advancement of the access sheath over the balloon), fragmentation time (defined as the time elapsed from insertion of the rigid nephroscope to the placement of the nephrostomy tube), estimated blood loss and total operative time (defined as the time elapsed from initial cystoscopy for ureteral catheter placement until the placement of the nephrostomy tube), and postoperative outcomes were also recorded in this study.

## 3. Results

From March to August 2015, thirty-eight patients underwent PCNL where ultrasound was used to guide all steps of renal access and tract dilation and were enrolled in our study. There were 17 males and 21 females with a mean age of 52.7 ± 17.2 years and American Society of Anesthesiologists (ASA) physical status classification ranged from class 1 to class 3. Stone size varied from 9.0 to 79.4 millimeters (mean 27.2 ± 17.6 millimeters) and only ten of these thirty-eight kidneys demonstrated more than mild hydronephrosis on preoperative imaging (Table 1). Compared to a fluoroscopic control group of 38 cases done by the same surgeon prior to the adoption of ultrasound guidance, both groups were comparable in terms of demographic and preoperative parameters. Body mass index (BMI) was the only difference of note where it was significantly lower in the ultrasound group (26.1 versus 30.3 kg/m^2^, *p* = 0.03).

Puncture time varied from 9 to 540 seconds with a mean of 135.4 ± 132.5 seconds (Table 1). Mean dilation and fragmentation times were 11.5 ± 3.8 and 37.5 ± 29.0 minutes, respectively. While the upper and lower calices were selected for puncture almost equally in the ultrasound group, the lower calyx was targeted most often in the fluoroscopic group. Mean total operative time was 129.3 ± 41.1 minutes, which was not statistically significantly different compared to a control of 147.1 ± 52.1 minutes for the fluoroscopic group. While no fluoroscopic screening was required during renal access or tract dilation, fluoroscopic imaging was used for confirmation of nephrostomy tube positioning at the end of the procedure in all cases. For these procedures, mean fluoroscopic screening time was 17.7 ± 13.3 seconds (range 1.0 to 63.0 seconds) and mean radiation exposure dose was 3.1 ± 3.2 mGy (range 0.2 to 14.0 mGy). Comparatively, in the fluoroscopic control group of patients, total fluoroscopic screening time and radiation exposure were 182.9 ± 119.0 seconds and 47.5 ± 52.3 mGy, respectively. Intraoperatively, all patients were visually and ultrasonographically confirmed stone-free, confirmed by KUB and ultrasound performed at 30-day follow-up.

One grade 2 complication as defined by the Clavien-Dindo classification was encountered during the immediate postoperative period [13]. This was a female patient with a history of recurrent UTI who underwent concurrent contralateral ureteroscopy and laser lithotripsy for a renal stone. She experienced prolonged fever postoperatively and was managed successfully with broad-spectrum antibiotics.

On the morning of the first postoperative day, creatinine was not statistically significantly changed compared to preoperative values. Change in hematocrit was not statistically significantly different between the ultrasound and fluoroscopic groups and no blood transfusions were necessary in either group. No additional complications were seen at follow-up of 30 days. No secondary procedures were required for all patients (Table 1).

## 4. Discussion

Percutaneous nephrolithotomy is the primary procedure used for the management of patients with large renal stones not amenable to ureteroscopy or shockwave lithotripsy. In the United States and around the world, fluoroscopic guidance has been the primary imaging method of choice used to guide percutaneous renal access and establish the working tract to facilitate these procedures. Over time, concerns have grown that long-term cumulative ionizing radiation exposure may possibly increase the incidence of malignancies [14–16]. For nephrolithiasis patients, reducing their exposure to ionizing radiation in all settings is an important goal as these patients are at high risk for increased cumulative radiation exposure compared to patient populations without nephrolithiasis [17]. Intraoperative radiation exposure to surgical staff not only results from direct exposure to the fluoroscopic radiation beam that is in close proximity to many staff members, it also stems from beam scatter produced from the interaction between the primary radiation beam with the patient and the operating table [4, 6]. While some have shown that during PCNL radiation exposure may be relatively low, practices may vary from place to place and case to case [5]. Despite usage of protective equipment, of all the operating room personnel, the surgeon generally receives the maximum ionizing radiation exposure, mostly to the legs and the eyes [6].

Several methods have been proposed to facilitate renal access while reducing radiation exposure for PCNL. One such alternative is endoscopic guidance, commonly known as Endoscopic Combined IntraRenal Surgery (ECIRS). This technique begins with retrograde access into the renal collecting system using a flexible ureteroscope. After positioning the ureteroscope in the target calyx of choice, fluoroscopy is used to guide a needle into the kidney in an antegrade percutaneous fashion and the needle enters the collecting system under direct vision of the ureteroscope. It can be performed either in a modified supine [18] or prone split-leg position [19, 20]. Once a target calyx is identified by flexible ureterorenoscopy, fluoroscopy is still needed for caliceal puncture. Compared to standard PCNL, however, this technique can be associated with lower fluoroscopic screening time and increased stone clearance [19, 21].

Real-time diagnostic ultrasonography (US) is becoming more widely accepted as an alternative imaging modality for directing PCNL in a dilated renal collecting system. The overall success rate can be comparable to that of standard fluoroscopic-guided PCNL [22]. Ultrasonography is free of ionizing radiation and effective and offers an advantage of portability compared to fluoroscopy. In addition, it provides additional imaging information over fluoroscopy during PCNL, including imaging and identification of viscera and structures that might be located between the skin and kidney, the depth of penetration of the puncture needle relative to the target calyx, and an easier means of differentiating posterior from anterior calyces. For these reasons, ultrasonography can help prevent adjacent and visceral organ injury such as inadvertent colonic penetration. In addition, there is no need for routine retrograde contrast or fluid injection and therefore the use of ultrasonography eliminates the need for a retrograde ureteral catheter if the surgeon is faced with the problem of unsuccessful retrograde ureteral catheterization [23]. Given its radiation-free nature, it is also an ideal imaging guidance modality for patient populations more sensitive to radiation exposure, including pediatric and pregnant patients [24]. Moreover, the use of US at the end of the procedure helps the urologist to look for nonopaque and semiopaque residual stones that are not visualized by radiography to confirm stone-free status intraoperatively [25].

In an attempt to minimize ionizing radiation exposure during PCNL, renal access with ultrasound guidance was first reported by Desai et al. in 1999. PCNL was done with US-guided percutaneous puncture in 45 renal units for 40 pediatric patients. However, fluoroscopic screening was still required during tract dilation. Overall stone clearance rate of 91% was achieved with few minor complications [26]. The largest published series of ultrasound-guided PCNL comes from Li et al. In 2014, they reported 8025 cases performed by a single surgeon [27] demonstrating a final stone-free rate of 85.5%. Yan et al. demonstrated a similar stone-free rate with their series of 679 patients [25]. Our series differs from all previously mentioned studies in that we demonstrate that ultrasound imaging can be used to reliably visualize and guide all steps of renal access and tract dilation without having to rely on fluoroscopic screening, supine positioning, or a two-step direct visualization method.

This is also, to our knowledge, the first series to describe that this technique and approach can be used to successfully gain renal access for PCNL in any degree of hydronephrosis. In our experience, while placement of a ureteral catheter and subsequent infusion of a small amount of normal saline could help identify calyces for puncture, we did not find that large volume injection was necessary. We were successful in gaining renal access even in the absence of moderate or severe hydronephrosis and all of our procedures were performed with the patient in the prone position. With modern ultrasound imaging consoles, the nondilated system can be visualized well even in the prone position, and it may be that improvement in imaging technology and quality since Desai's first publication has made an ultrasound-guided approach to PCNL more feasible and less risky for patients.

Using ultrasound to guide PCNL is not without its technical challenges, however. In our practice, a high-pressure balloon dilator is routinely used for tract establishment. One difficulty with accurate placement of the balloon with ultrasonography is that the deflated balloon tip is not easily visualized under ultrasound guidance. Once inflated, the balloon can readily be seen, but its tip can be difficult to accurately identify in the deflated state. We overcame this problem by constantly moving the wire back and forth while passing the balloon over it and looking for a change in the wire contour to judge where the tip of the balloon was relative to the wire. Despite this, the tip of the balloon could still be difficult to distinctly visualize. In addition, since ultrasound imaging relies on real-time movement of the probe over the patient's body and the probe is not fixed in place, one technical challenge presented is the need to keep the ultrasound probe on the operative field while passing the wire and dilation instruments into the kidney. With the technology currently available, these multiple tasks require at minimum two sets of hands in order to be performed efficiently. As demonstrated in Figures 1–3, we standardized our procedures to minimize the amount of assistance required by the primary surgeon by having one assistant stand behind the surgeon toward the foot of the bed. In this fashion the operative surgeon performed all steps of renal access and tract dilation while holding the ultrasound probe with the nondominant hand in place over the kidney while the assistant held control of the wire to facilitate dilation. Accomplishing this, however, requires a coordinated team effort. Lastly, with ultrasound imaging, the needle, wire, and dilators can readily be imaged but the nephrostomy tube can often be difficult to visualize, highlighting an area of possible future technology development For this reason, we used limited fluoroscopic imaging to confirm adequate placement of the nephrostomy tube at the end of each case, as it was difficult to perform accurately with ultrasound imaging guidance alone.

Two significant differences between our cohorts that warrant discussion were a lower BMI in the ultrasound group and a higher use of the lower pole in the fluoroscopic group. These differences may have influenced our results. For example, our clinical outcomes, including stone-free and complication rates may have been higher in the ultrasound group had they been comprised of patients with a higher BMI. On the other hand, the higher rate of upper pole entry in the ultrasound group may have increased the likelihood of a complication such as pneumothorax in this group, though these differences were not seen. In balance, we appeared to perform both techniques with similar associated complication rates despite these differences. These results may therefore be more likely reflective of surgeon ability as opposed to patient characteristics. The best way to know this with greater certainty would be to perform a randomized trial for surgical technique, which was not the aim of this current study. While our ultrasound cohort is relatively small and consists of patients with a relatively low BMI, we present this series as a prospective, descriptive study in the hopes that the practicing urologist will be encouraged to utilize intraoperative ultrasonography for all steps of renal tract access and dilation during PCNL. In our series, the operative surgeon transitioned from fluoroscopic to ultrasound image guidance by performing a limited number of mentored cases over a short period of time. In this short period of time, applying ultrasound guidance to all steps of renal access and dilation was associated with a significant reduction in radiation exposure for intraoperative personnel and the patient. Particularly for the urologist who relies on fluoroscopy to guide percutaneous renal stone surgery, the techniques described with our experience may have significant relevance.

## 5. Conclusions

Ultrasound-guided technique for renal access and tract dilation in prone percutaneous nephrolithotomy is feasible and effective in treating renal stones. It can be performed successfully and safely even in the patients with a minimally dilated collecting system in the prone position. Our experience demonstrates good surgical outcomes with significant reduction in fluoroscopic screening time to the patient, surgeon, and intraoperative personnel.

## Figures and Tables

**Table 1:** Patient characteristics and perioperative parameters.

Parameter	Ultrasound group (*n* = 38)	Fluoroscopy group (*n* = 38)	*p* value
Preoperative characteristics			
Mean (SD) age	52.7 ± 17.2	52.9 ± 14.3	0.96
Gender, *n* (%)			
Male	17 (44.7)	19 (50.0)	0.65
Female	21 (55.3)	19 (50.0)	
Mean (SD) BMI (kg/m^2^)	26.1 ± 7.3	30.3 ± 8.2	0.03
Mean (SD) preoperative serum creatinine (mg/dL)	0.92 ± 0.33	0.97 ± 0.37	0.51
Mean (SD) preoperative hematocrit (%)	39.0 ± 5.9	40.1 ± 4.9	0.38
ASA physical status, *n* (%)			
Class 1	7 (18.4)	6 (15.8)	0.36
Class 2	17 (44.7)	23 (60.5)	
Class 3	14 (36.9)	9 (23.7)	
Stone laterality, *n* (%)			
Right	15 (39.5)	16 (42.1)	0.82
Left	23 (60.5)	22 (57.9)	
Stone type and position, *n* (%)			
Calyceal renal stone	12 (31.6)	9 (23.7)	0.24
Renal pelvic stone	10 (26.3)	14 (36.8)	
Staghorn stone	6 (15.8)	6 (15.8)	
Proximal ureteral stone	3 (7.9)	7 (18.4)	
Multiple stones	7 (18.4)	2 (5.3)	
Mean (SD) stone size (millimeters)	27.2 ± 17.6	28.5 ± 14.5	0.72
Degree of hydronephrosis, *n* (%)			
None	20 (52.6)	15 (39.5)	0.59
Mild	8 (21.1)	13 (34.2)	
Moderate	8 (21.1)	8 (21.1)	
Severe	2 (5.2)	2 (5.2)	

Intraoperative measurements			
Mean (SD) puncture time (seconds)	135.4 ± 132.5	NA	
Puncture location, *n* (%)			
Upper calyx	16 (42.1)	3 (7.9)	0.00
Middle calyx	7 (18.4)	1 (2.6)	
Lower calyx	15 (39.5)	34 (89.5)	
Mean (SD) dilation time (minutes)	11.5 ± 3.8	NA	
Mean (SD) fragmentation time (minutes)	37.5 ± 29.0	NA	
Mean (SD) total operative time (minutes)	129.3 ± 41.1	147.1 ± 52.1	0.11
Mean (SD) radiation exposure dose (mGy)	3.1 ± 3.2	47.5 ± 52.3	0.00
Mean (SD) fluoroscopic screening time (seconds)	17.7 ± 13.3	182.9 ± 119.0	0.00

Postoperative outcomes			
Mean (SD) postoperative serum creatinine (mg/dL)	0.92 ± 0.37	1.01 ± 0.39	0.33
Mean (SD) difference in preoperative and postoperative hematocrit (%)	4.0 ± 5.6	2.6 ± 3.5	0.18
Mean (SD) hospital stay (days)	3.1 ± 1.4	2.9 ± 1.9	0.67
Postoperative complication, *n* (%)			
Grade 1	0	0	0.56
Grade 2	1 (2.6)	0	
Grade 3	0	2 (5.3)	
Stone-free status, *n* (%)			
Stone-free	38 (100)	34 (89.4)	0.12
Insignificant residual stone	0	2 (5.3)	
Significant residual stone	0	2 (5.3)	
